# Retina-Inspired Organic Heterojunction-Based Optoelectronic Synapses for Artificial Visual Systems

**DOI:** 10.34133/2021/7131895

**Published:** 2021-02-22

**Authors:** Junyao Zhang, Yang Lu, Shilei Dai, Ruizhi Wang, Dandan Hao, Shiqi Zhang, Lize Xiong, Jia Huang

**Affiliations:** ^1^Interdisciplinary Materials Research Center, School of Materials Science and Engineering, Shanghai Institute of Intelligent Science and Technology, Tongji University, Shanghai 201804, China; ^2^Translational Research Institute of Brain and Brain-Like Intelligence, Shanghai Fourth People's Hospital Affiliated to Tongji University, Tongji University, Shanghai 200434, China

## Abstract

For the realization of retina-inspired neuromorphic visual systems which simulate basic functions of human visual systems, optoelectronic synapses capable of combining perceiving, processing, and memorizing in a single device have attracted immense interests. Here, optoelectronic synaptic transistors based on tris(2-phenylpyridine) iridium (Ir(ppy)_3_) and poly(3,3-didodecylquarterthiophene) (PQT-12) heterojunction structure are presented. The organic heterojunction serves as a basis for distinctive synaptic characteristics under different wavelengths of light. Furthermore, synaptic transistor arrays are fabricated to demonstrate their optical perception efficiency and color recognition capability under multiple illuminating conditions. The wavelength-tunability of synaptic behaviors further enables the mimicry of mood-modulated visual learning and memorizing processes of humans. More significantly, the computational dynamics of neurons of synaptic outputs including associated learning and optical logic functions can be successfully demonstrated on the presented devices. This work may locate the stage for future studies on optoelectronic synaptic devices toward the implementation of artificial visual systems.

## 1. Introduction

Generally, human beings sense a large number of stimuli from the complicated environment, such as light, pressure, chemicals, and sounds [1]. Nearly 80% of the obtained information, among these external stimuli, is detected through human visual systems [2, 3]. Hence, retinas are supposed to be one of the most significant sensory organs in human bodies, which have stimulated the emergence of artificial vision by bioinspired electronics [4–7]. Additionally, towards the future development of humanoid soft robots and artificial intelligence systems, there is a tremendous demand to design advanced photoelectric detection devices capable of being operated under various ambient conditions to strengthen and ultimately substitute for human visual systems in the field of industry, science, and military [8–11]. In order to mimic the visual perception of biological retinas, artificial visual systems composed of perceiving, processing, and memorizing capabilities have been inspired.

Optoelectronic synapses, which allow the integration of information sensing, processing, and memorizing in a single device, have become a promising candidate for developing artificial visual systems [12–15]. They have a more compact architecture than the traditional one that is composed of two devices, and offer capabilities beyond simple sensing devices. So far, although diverse kinds of optoelectronic synapses including memristors [2, 6, 16–19], and field-effect transistors [4, 20–28], have been extensively demonstrated, most reported optoelectronic synapses have focused on the simulation of basic synaptic behaviors by specific wavelength. Human retinas are responsible for recognizing and detecting different wavelengths of visible light, so it is extraordinarily meaningful to realize the mimicry of color recognition and visual-aided learning in artificial visual systems. Only a few studies have reported on artificial synaptic devices of retinal-like functions, while part of them separated optical-sensing and synaptic functions into two devices [5, 9], and others could only recognize two different wavelengths of light [3, 29, 30]. Besides, compared to memristors, field-effect transistors are more promising for linear plasticity, which is a key factor for the effect of image learning and recognition [12, 13]. Therefore, by simulating biological retinas, constructing optoelectronic synaptic transistors that incorporate the synaptic and sensing elements in a single unit for color recognition and visual-aided learning will expand the potential and application of artificial visual systems.

Here, we introduced optoelectronic synaptic transistors based on organic heterojunction that exhibited color recognition and functions of effectively perceiving, processing, and memorizing the information in a single device. The heterojunction consisted of poly(3,3-didodecylquarterthiophene) (PQT-12) and tris(2-phenylpyridine) iridium (Ir(ppy)_3_) layers. Through the modulation of heterojunction, some typical synaptic functions, such as excitatory postsynaptic current (EPSC), paired-pulse facilitation (PPF), and the transition from short-term memory (STM) to long-term memory (LTM), were all successfully simulated. Under different wavelengths of light, distinct synaptic characteristics could be achieved. More importantly, synaptic transistor arrays were constructed to demonstrate their optical perception efficiency under diverse illuminating conditions and color recognition capability. The wavelength-tunability of synaptic behaviors further enabled the mimicry of visual learning and memorizing processes of humans under different mood states. Finally, computational dynamics of neurons of synaptic outputs, including associated learning and optical logic functions, could be emulated on the optoelectronic synapses. Such a presentation of the organic heterojunction-based optoelectronic synaptic transistors can promote the development of novel devices for future artificial visual systems.

## 2. Results and Discussion

**Figure 1:**
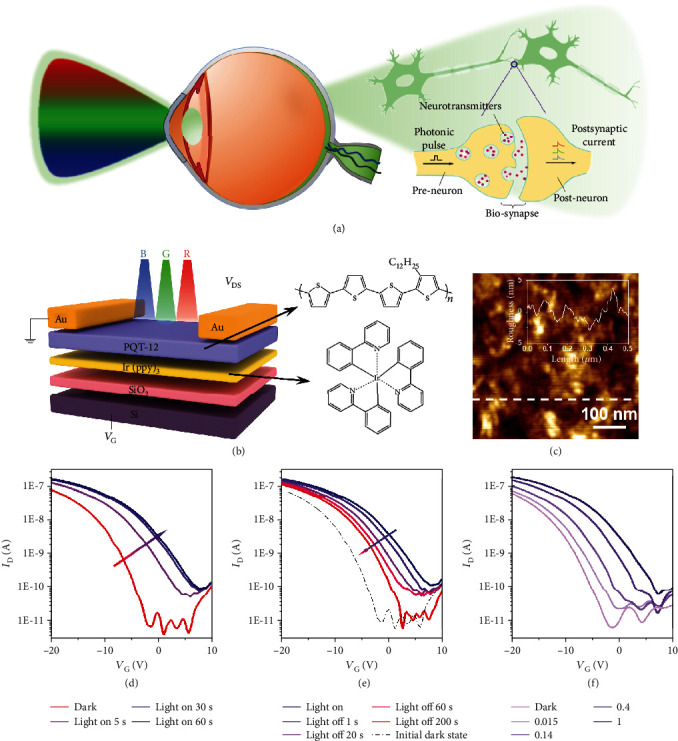
Overview of a human visual system and the PQT-12/Ir(ppy)_3_ optoelectronic synapse. (a) Schematics of a human visual system. (b) Device structure of PQT-12/Ir(ppy)_3_ optoelectronic synapse modulated by multiple wavelengths of light and molecular structures of PQT-12 and Ir(ppy)_3_. (c) AFM image of the PQT-12/Ir(ppy)_3_ film on Si/SiO_2_ substrate. The insert is the roughness curve of the PQT-12/Ir(ppy)_3_ film surface. Transfer characteristics curves of the PQT-12/Ir(ppy)_3_ optoelectronic synaptic transistor with different (d) light-on and (e) light-off times with fixed wavelength of 540 nm at a constant *V*_D_ of -10 V. (f) Transfer characteristics curves of the PQT-12/Ir(ppy)_3_ optoelectronic synaptic transistor with a fixed wavelength of 540 nm and different light densities (mW cm^−2^) at a constant *V*_D_ of -10 V.

The human brain receives perceptual information through sensory organs for cognitive processing [1, 5]. For light signals, human beings own cone cells to detect and recognize information from different wavelengths of visible light, and then perform information preprocess before continuing to transmit information to the visual cortex of the brain (Figure 1(a)). Synapse is the functional link between two neurons [31, 32]. The release of neurotransmitters from the presynaptic neuron is triggered upon the arrival of light stimuli. These neurotransmitters are eventually received by appropriate receptors in the postsynaptic membrane, triggering a postsynaptic potential [33]. Thus, the postsynaptic current (PSC) is ultimately generated, which is utilized to assess the connection strength of the synapse. To simulate the functions of human retinas, optoelectronic synaptic transistors based on PQT-12/Ir(ppy)_3_ organic heterojunction were constructed (Figure 1(b)). The fabrication of our synaptic transistors is introduced in detail in the Materials and Methods. Figure S1 shows the cross-sectional scanning electron microscope (SEM) of the as-fabricated optoelectronic synaptic transistors. The light stimuli and the channel layer of the synaptic transistors were served as the presynaptic and postsynaptic membrane, respectively. The PQT-12/Ir(ppy)_3_ organic heterojunction was sensitive to light, therefore, laying the foundation for modulating the conductivity of the channel. PQT-12 was responsible for the carrier transport, which exhibited high photoresponse to visible light [15]. Ir(ppy)_3_ that attracted tremendous interest in OLED owing to its high efficiency of electrophosphorescence was used to form the heterojunction structure and served as the electron-trapping layer [34, 35]. The surface morphology of Ir(ppy)_3_ film and PQT-12 on Ir(ppy)_3_ film was investigated through atomic force microscopy (AFM), as shown in Figure 1(c) and Figure S2. The root mean square roughness (RMS) of Ir(ppy)_3_ film and PQT-12 on Ir(ppy)_3_ film was found to be 0.35 and 1.33 nm, respectively, and the roughness at different positions was pretty low, which confirmed the inappreciable device-to-device variation and the uniformity of our subsequent synaptic arrays. The typical output characteristics of the synaptic transistors were measured in the dark condition, as depicted in Figure S3, and decent organic transistor performance was exhibited. Once the optoelectronic synaptic transistor was upon the irradiation of light, the positive shift of the transfer curve could be observed, and as the exposure time extended, the positive displacement of the transfer curve was also increased (Figure 1(d)). Positive shift tended to be saturated when the irradiation time exceeded a certain time. After the optoelectronic synaptic transistor was illuminated for 60 s, the light source was removed, leading to the negative shift of the transfer curve. Even when the light was off for 200 s, compared to the original dark condition, a certain positive shift was still demonstrated on the transfer curve (Figure 1(e)). Figure 1(f) illustrates the transfer characteristics curves under different illumination intensities. Although the illumination intensity was low to 0.015 mW cm^−2^, the optoelectronic synapse could still detect it, confirming the high photoresponsivity of PQT-12/Ir(ppy)_3_ heterojunction. It should be noted that during the storage process and before the testing process, the device would inevitably be illuminated. Thus, the photogenerated electrons could retain in Ir(ppy)_3_ and the interface of the heterojunction, which resulted in the slightly positive shift of the transfer curve under the original dark condition.

The organic heterojunction-based optoelectronic synapse showed diverse synaptic characteristics under the light illumination. Figure S4 demonstrates a typical EPSC behavior induced by a presynaptic light pulse. The measured EPSC reached its maximum value of ∼-1.07 nA and then gradually decayed back to the resting current, which was similar to the change of the EPSC in a biological synapse [36]. PPF is a dynamic enhancement of neurotransmitter release, where the facilitation effect of presynaptic triggered EPSC decreases as the interval time (Δ*t*) between two consecutive pulses increases, which is regarded to be critical in the processing of a biological synapse [37]. Figure S5a describes the device responses by a pair of consecutive light pulses with a duration of 250 ms. The EPSC induced by the second light pulse (*A*_2_) was higher than that induced by the first light pulse (*A*_1_), which was due to the fact that the second light pulse was exerted before the EPSC triggered by the first one had completely decayed to the original state. Figure S5b shows the PPF index (*A*_2_/*A*_1_) as a function of Δ*t*. The PPF index exponentially decayed as Δ*t* increased, and this response demonstrated that the optoelectronic synapses had stable short-term plasticity characteristics.

**Figure 2:**
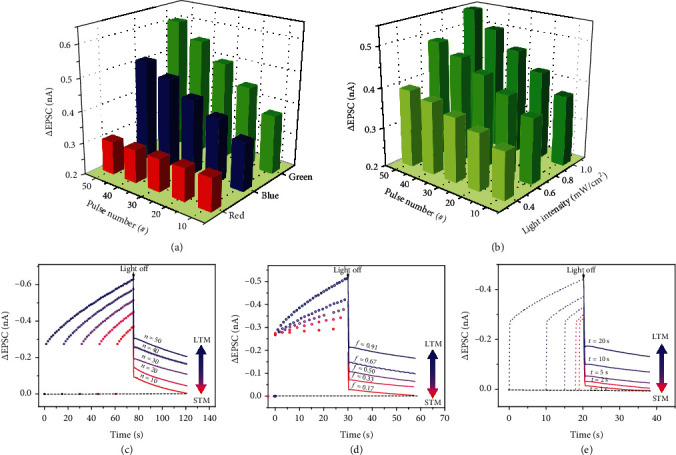
Output characteristics of the optoelectronic synapse. EPSC changes of the optoelectronic synapse induced by light pulses (a) under different wavelengths but an identical intensity of 0.66 mW cm^−2^ and (b) under green light with various densities (1 s duration with 0.5 s interval). The STM-to-LTM transition triggered by increasing the (c) number, (d) frequency, and (e) illuminating time of light pulses.

When three colors of light pulses (wavelengths: blue, 480 nm; green, 540 nm; red, 665 nm) were applied to the PQT-12/Ir(ppy)_3_ optoelectronic synapses, respectively, different EPSC responses were observed (Figure 2(a) and Figure S6). Under the illumination of a series of blue light and green light pulses, the photon energy was larger than the bandgap of PQT-12, and as a result, the photogenerated holes and electrons were generated. Due to the built-in electric field between PQT-12 and Ir(ppy)_3_, a great number of photogenerated electrons effectively moved into Ir(ppy)_3_ and the interface of the heterojunction, and thus, the photocurrent was triggered, which resulted in the positive shift of the transfer curve under the light illumination. This phenomenon could also be confirmed by the steady-state photoluminescence (PL) measurement (Figure S7). Compared with the PL intensity of pure Ir(ppy)_3_ thin film, the PL intensity of PQT-12/Ir(ppy)_3_ thin film was much weaker, suggesting through the heterojunction structure, photogenerated holes and electrons had been efficiently separated. After the light pulses were ended, the photogenerated carriers retaining in PQT-12 rapidly recombined with each other, which resulted in the first rapid decay of the EPSC. Since photogenerated electrons transferred into Ir(ppy)_3_ and the interface of the heterojunction were split from photogenerated holes remaining in PQT-12 spatially, they had a prolonged lifetime, primarily causing the slow decay of the EPSC in the next step [3, 15, 24]. When the green light pulses were exerted, a larger EPSC was observed compared with that triggered by blue light pulses under the same light pulse intensity and pulse time. This might be due to the higher separation efficiency of photogenerated carriers below green light than that below blue light. Under the illumination of a series of red light pulses, a much smaller peak EPSC value was achieved than that in previous cases. After the light pulses were removed, the EPSC nearly decayed to the origin state quickly, and increasing the number of red light pulses hardly affected the EPSC. This was because the incident photons had less energy than the bandgap of PQT-12, and as a result, only impurity levels in PQT-12 would absorb the photons and cause impurity-to-band transition, resulting in a rather weak EPSC [38]. Since the photogenerated carriers retaining in PQT-12 could rapidly recombine with each other, the EPSC barely increased after the illumination of a series of red light pulses. It should be noticed that in our synaptic device, octyltrichlorosilane- (OTS-) pretreated silica was utilized as the dielectric layer to suppress the charge trapping effect caused by the dielectric/semiconductor interface. In order to further verify the operating principle of the EPSC responses, a transistor based on pure PQT-12 was fabricated. As shown in Figure S8, the pure PQT-12-based transistor presented a quite weak photosensitivity under green light and the EPSC under red light pulses immediately returned to its initial state when the light source was removed. This also indicates the PQT-12/Ir(ppy)_3_ heterojunction was high photoresponsivity and was responsible for the EPSC responses. Thus, our optoelectronic synaptic transistors could be effectively utilized for color recognition even if the received light intensity of different colors was different. Different EPSC responses were observed when three colors of light pulses were applied to the PQT-12/Ir(ppy)_3_ optoelectronic synapses. The EPSC responses had distinguished fast and slow decay processes under different colors of light, which were enough to realize color recognition.

Moreover, to further identify photosynaptic responses, the device was then illuminated to the green light pulses with different intensities and durations. As shown in Figure 2(b) and Figure S9, a larger EPSC could be achieved through either a higher light intensity or a longer exposure time. In neurophysiology, there are two types of memory behaviors, STM and LTM [36]. By employing repeated pulse stimuli that continuously enhance the synaptic strength, the STM will be transferred to the LTM (Figure S10). To emulate the transition from STM to LTM, light pulses with varied numbers were applied to the optoelectronic synapse (Figure 2(c)). The EPSC change increased from -0.37 to -0.63 nA with the increase of pulse numbers from 10 to 50, which suggested a stronger memory level. The decay time was also proportional to the pulse numbers, indicating a longer memory retention time. Similarly, the transition from STM to LTM could be effectively realized by the modulation of frequency, duration time, and illumination intensity of the light stimuli, as depicted in Figures 2(d) and 2(e), and Figure S11, respectively. With the increase in frequency, duration, or illumination intensity, learning effectiveness would be gradually changed from low to high. LTM behavior was further studied by applying a light pulse with a width of 30 s (Figure S12). After the pulse was removed, the EPSC could retain over the initial level for nearly 900 s, which proved the excellent retention capability of the device.

In biological systems, the dynamic filtering capability is essential in signal processing [39, 40]. Figure S13a demonstrates the schematic illustration of the high-pass filtering function of biological synapses. The input signals with a frequency lower than the cutoff frequency (*f*_c_) will be enormously weakened, while the signals with a frequency higher than *f*_c_ will be permitted to pass through. Figure S13b presents the EPSC response after 30 successive light pulses. The thirtieth EPSC change was much larger than that of the first one. The EPSC gain (defined as *A*_30_/*A*_1_) was enhanced with the increase of pulse frequency (Figure S13c). It is also observed that there was an approximately linear relationship between the EPSC gain and presynaptic pulse frequency, suggesting the potential of the optoelectronic synapse served as a high-pass filter for information processing.

**Figure 3:**
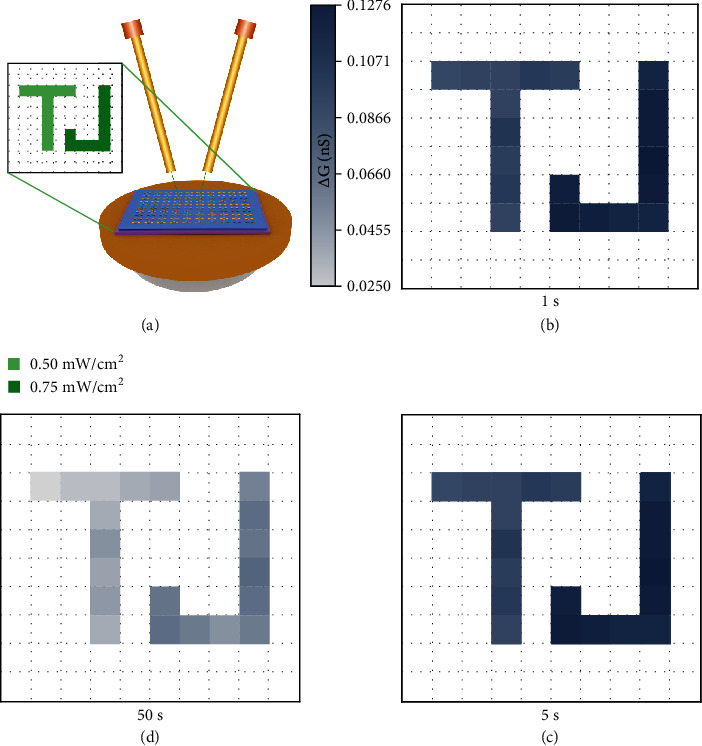
A demonstration of the 10 × 10 synaptic transistor array. (a) Schematic illustration of the basic operating principle of the 10 × 10 synaptic transistor array under the illumination of different light intensities at a fixed wavelength of 540 nm. ∆*G* of the devices in the 10 × 10 transistor array after the illuminating process in (b) 1 s, (c) 5 s, and (d) 50 s.

With the integrated signal sensing, processing, and memorizing functions under different illuminating conditions, our optoelectronic synapses are suitable to mimic the visual perception of the retina. A synaptic transistor array with10 × 10 devices was constructed, and the operating schematic diagram was demonstrated in Figure 3(a). The certain synaptic transistor units in the array were exposed to 20 consecutive green light pulse signals under a certain intensity (“*T*”: 0.50 mW cm^−2^, and “*J*”: 0.75 mW cm^−2^). Channel conductance change (*∆G*) can be considered as the memory level. According to Figure 3(b), two letters of “*T*” and “*J*” were encoded and recognized through the light intensity. Under the illumination of high light intensity (the letter “*J*”), more photogenerated electrons moved into Ir(ppy)_3_ and the interface of the heterojunction and thus resulted in larger *∆G*, while fewer photogenerated electrons moved into Ir(ppy)_3_ and the interface of the heterojunction under low light intensity (the letter “*T*”). And as time went on, both “*T*” and “*J*” were still memorized and possessed the ability to be recognized (Figures 3(c) and 3(d)).

**Figure 4:**
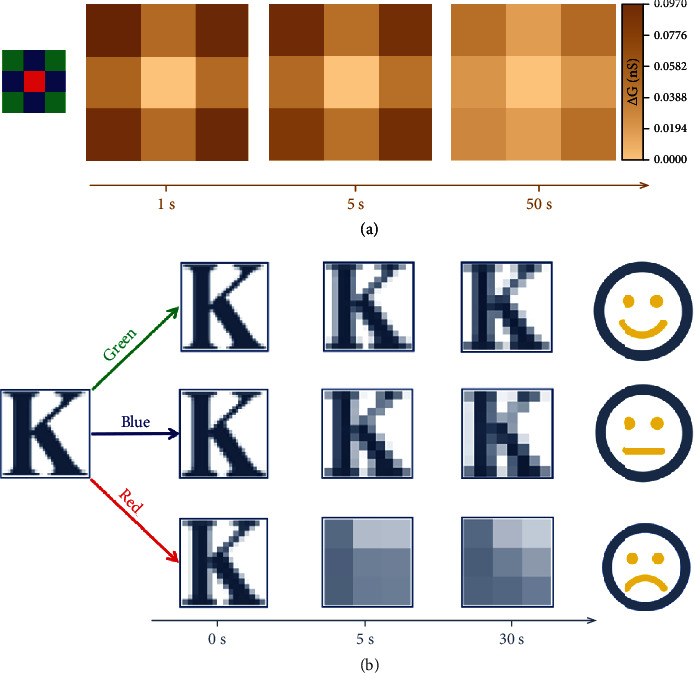
A demonstration of the 3 × 3 synaptic transistor array and the mimicry of human visual learning and forgetting processes. (a) Schematic illustration of the basic operating principle of the 3 × 3 synaptic transistor array under the illumination of different light wavelengths (480 nm, 540 nm, 650 nm) with a fixed light intensity of 0.5 mW cm^−2^, and the corresponding test results of the transistor array after the illuminating process in 1 s, 5 s, and 50 s. (b) Mimicry of human visual learning and forgetting processes in different mood states, and the recognition result of the letter “*K*” denoted by the light wavelengths of 480 nm (blue), 540 nm (green), and 650 nm (red), respectively.

Furthermore, nine optoelectronic synaptic transistors were selected from the above array randomly to investigate the capability of color recognition by an input image composed of 3 × 3 pixels. Each optoelectronic synapse was applied to 20 successive light pulses with a fixed light intensity under different wavelengths of visible light (blue, green, red) based on the target image. As depicted in Figure 4(a), the target image was successfully recognized and memorized according to the *∆G* contrast. Similarly, the image could be precisely identified by various colors of light over time, and even after 50 s, the difference of *∆G* was still enough to distinguish different colors. As a result, our optoelectronic synaptic transistors were most sensitive to green light, next to blue light, and least sensitive to red light, which were similar to the characteristics of human retinas. Although the current retina-inspired devices could only imitate part of the dynamic range and functions of human retinas, they also promoted the development of building future artificial visual systems.

Obviously, emotion can affect the learning, memorizing, and forgetting processes [41–43]. Here, as a demonstration, our heterojunction-based optoelectronic synapses were revealed to simulate the learning and memorizing processes of humans in different moods that were represented by the light of different colors. It can be defined that higher *∆G* corresponds to a higher learning level. There is a positive correlation between learning level and mood, so green light is defined as a good mood, due to the highest *∆G* achieved under green light according to the previous results. In addition, it is observed that under a good mood, the memorized results will be forgotten more slowly. On this account, blue and red light are deemed as neural and bad moods, respectively. The input letter image was divided into 1024 squares.  Figure 4(b) shows the memorizing and forgetting processes under different moods through the simulation of letter image recognition and learning. When a person possessed a bad mood, only a relatively vague impression of the letter “*K*” learning level was obtained (0 s). While a clearer impression of “*K*” could be achieved when a person had a good mood. After learning as time went by, the memory level on “*K*” under a better mood would also be higher, and even after 30 s, the “*K*” could still be identified. On the contrary, the memory level on “*K*” under a bad mood would soon return to its original state. This confirms the suitability of using our device to construct an artificial visual system.

**Figure 5:**
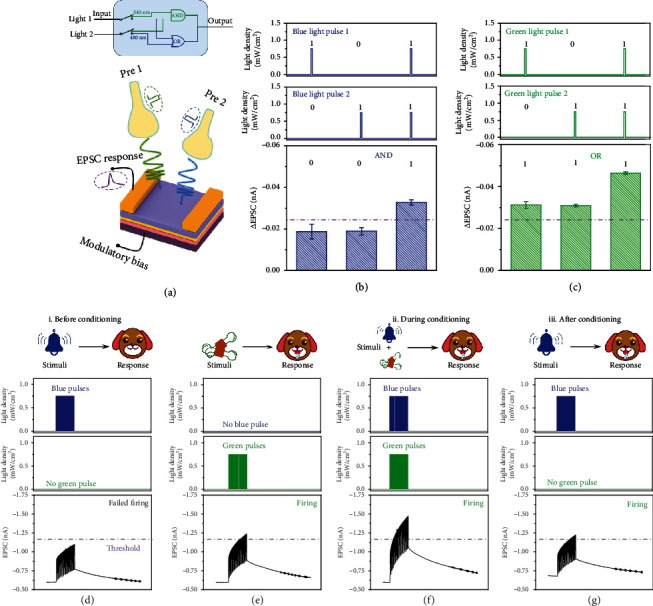
Optical logic functions and associated learning on the optoelectronic synapse. (a) The schematic diagram of optical logic functions of “AND” and “OR” operations. The input (presynapse1 (pre 1) and presynapse2 (pre 2)) and output (∆EPSC) characteristics of the (b) “AND” operation and (c) “OR” operation. (d)–(g) Imitation of classical Pavlov's learning by using all-optical inputs of 480 nm wavelength as bell ringing/conditioned stimuli and 540 nm wavelength as food/unconditioned stimuli. The duration and power density of each light pulse are 1 s and 0.75 mW cm^−2^, respectively. The interval between the pulses is 0.5 s. The purple dash-line represented the threshold for salivation is set at -1.15 nA.

Computational dynamics of neurons of synaptic outputs, including logic functions and associated learning [44], are accomplished through all-optical stimulation. First, optical logic functions of “AND” and “OR” operations were explored in our device by introducing the illumination of blue light and green light with the same light intensity and illuminating time. The schematic diagram of the “AND” and “OR” logic operation switching under different wavelengths is illustrated in Figure 5(a). The change of EPSC (*∆*EPSC) one second after the stimulus was removed was employed as the output signal of the device, and *∆*EPSC of -0.025 nA was defined as the threshold. Under the illumination of blue light, individual optical pulse demonstrated weak ∆EPSC, below the threshold (level “0”). Only when two light pulses were performed simultaneously, the ∆EPSC triggered was larger than the value of the threshold, which was deemed as the “AND” logic operation (Figure 5(b)). While for the illumination of green light, either light pulse 1 or light pulse 2 was applied, the ∆EPSC triggered was over the value of the threshold, which was treated as the “OR” logic operation (Figure 5(c)). Thus, the “AND” and “OR” logic operations were readily converted by utilizing different wavelengths of light, demonstrating a type of emulation for neuronal computation in neuromorphic systems. In addition to modulating the wavelengths of light, optical logic functions of “AND” and “OR” operations were also accomplished in the optoelectronic synaptic transistors through the illumination of blue light with different light intensities (Figure S14).

Utilizing the distinct absorption characteristics of different wavelengths of visible light, the classical Pavlovian conditioning [44, 45], also known as an example of associative learning, was mimicked in our optoelectronic synapses. Here, stimulation with 20 consecutive blue light pulses was considered as bell ringing/conditioned stimuli while stimulation with 20 consecutive green light pulses was considered as food/unconditioned stimuli. In our experiment, EPSC of -1.15 nA was defined as the threshold for salivation set. As shown in Figure 5(d), when 20 bell ring stimuli were exerted to the device, the EPSC was always under the salivation threshold after repetitive rehearsal, while 20 food stimuli induced relatively higher EPSC than that induced by the bell ring stimuli, which was over -1.15 nA, illustrating salivation response was triggered (Figure 5(e)). After simultaneous stimulation with bell ringing and food, the EPSC response was larger than that caused by the previous individual stimuli, indicating that an associative reflex was built between unconditioned and conditioned stimuli (Figure 5(f)). And then, the EPSC above the salivation threshold was achieved only by bell ringing sequence (Figure 5(g)), which successfully emulated the salivation response of the Pavlov's dog on bell ringing stimuli. After our optoelectronic synapses were illuminated by both bell ringing and food stimulation, more photogenerated electrons entered Ir(ppy)_3_ and the interface of the heterojunction, which played a significant role in the associative learning.

## 3. Conclusion

In summary, we have demonstrated optoelectronic synaptic transistors based on organic heterojunction that exhibit color recognition and the functions of effectively perceiving, processing, and memorizing the information in a single device. The organic heterojunction serves as a basis for distinctive synaptic characteristics under different wavelengths of light. Through the modulation of heterojunction, some basic synaptic functions, such as EPSC, PPF, and the transition from STM to LTM, are all successfully simulated. Under different wavelengths of light, distinct synaptic characteristics can be achieved. Besides, synaptic transistor arrays are constructed to demonstrate their optical perception efficiency under diverse illuminating conditions and color recognition capability. The wavelength-tunability of synaptic behaviors further enables the mimicry of mood-modulated visual learning and memorizing processes of the human. More importantly, computational dynamics of neurons of synaptic outputs including associated learning and optical logic functions are also emulated. This work provides the potential for the application of retina-inspired optoelectronic synapses to realize artificial visual systems, which remains challenging in the reality.

## 4. Materials and Methods

### 4.1. Materials and Device Fabrication

Poly(3,3-didodecylquarterthiophene) (PQT-12) with a molecular weight of 16000 and tris(2-phenylpyridine) iridium (Ir(ppy)_3_) were purchased from J&K. All chemicals were used as received without further purification.

Devices were made using a heavily n-doped silicon wafer with 100 nm thermally grown SiO_2_ as substrate. The substrate was ultrasonically cleaned by acetone and 2-propanol for 30 min, respectively. After that, the substrate was rinsed by ethanol and distilled water, dried by nitrogen flow, and then put in an oven at 60°C to dry off. The washed substrate was submerged into the OTS solution in hexane and chloroform to form a self-assemble layer. Afterward, 10 nm Ir(ppy)_3_ film was thermally evaporated onto the OTS-pretreated silica. Then, PQT-12 film was prepared by spin coating PQT-12 solution (3 mg/ml, chloroform) at a speed of 2000 rpm for 40 s and annealing (100°C, 30 min) in the glovebox. 50 nm Au electrodes were thermally evaporated onto the top of the PQT-12 film through shadow masks with a channel length (L) of 30 *μ*m and width (W) of 1 mm. The synaptic transistor arrays were also fabricated by the same process on a silicon wafer substrate.

### 4.2. Device Characterization

The surface morphology of Ir(ppy)_3_ film and PQT-12 on Ir(ppy)_3_ film was investigated by atomic force microscopy (AFM, SEIKO SPA-300HV). The cross-sectional image of the device was achieved by a scanning electron microscope (SEM, Nova Nano SEM 450). The PL spectra of the films were obtained from A F-7000 spectrophotometer (Hitachi). All electrical performances were measured by Keithley 4200 SCS in the air at room temperature. Xenon lamps and double grating monochromators (Omno 330150, Beijing NBeT, China) were utilized to offer a light source with a fixed wavelength and different intensities.

### 4.3. Simulation

The letter recognition under different moods was emulated with the assistance of the MATLAB. The image of letter “*K*” was treated as an example, which was divided into 1024 squares. It is assumed that the maximum value of EPSC triggered by twenty light pulses corresponded to all squares identified. By considering the ratio of the real-time EPSC to the maximum EPSC, the number of the recognized squares was achieved. As the ratio increased, the quantity of the recognized squares increased, and the picture identified would be clearer.

## Data Availability

All data needed to evaluate the conclusions in the paper are presented in the paper and/or the Supplementary Materials. Additional data related to this paper may be requested from the authors.

## Supplementary Materials

Supplementary Materials Figure S1. Cross-sectional SEM image of the optoelectronic synaptic transistor based on PQT-12/Ir(ppy)3 organic heterojunction. Figure S2: AFM images of the Ir(ppy)3 film and PQT-12/Ir(ppy)3 film on Si/SiO2 substrate at different positions. Figure S3: The output characteristics curves of the optoelectronic synaptic transistor in the dark condition with different VG from 0 to -10 V. Figure S4: The EPSC behavior of the optoelectronic synapse. Figure S5: The PPF behavior and the corresponding PPF index curve of the optoelectronic synapse. Figure S6: The EPSC triggered by light pulses under different wavelengths. Figure S7: Energy band diagram and the steady-state PL spectrum of PQT-12/Ir(ppy)3 heterojunction. Figure S8: The transfer characteristics curves and light switch performance of the pure PQT-12 transistor. Figure S9: The EPSC triggered by light pulses under different light intensities. Figure S10: Schematic diagram of the biological memory consolidation process in the human brain. Figure S11: The STM-to-LTM transition triggered by increasing the illumination intensity of light pulses. Figure S12: LTM behavior of the optoelectronic synaptic transistor. Figure S13: The demonstration of high-pass filtering function of the optoelectronic synapse. Figure S14: Optical logic functions on the optoelectronic synapse through the illumination of blue light with different light intensities.
