# Supplementary material for: Retina-Inspired Organic Heterojunction-Based Optoelectronic Synapses for Artificial Visual Systems
Source: Research (Wash D C). 2021 Feb 22;2021:7131895. doi: 10.34133/2021/7131895 (PMC7926506; doi:10.34133/2021/7131895)
Supplement: Supplementary Materials — Figure S1. Cross-sectional SEM image of the optoelectronic synaptic transistor based on PQT-12/Ir(ppy)3 organic heterojunction. Figure S2: AFM images of the Ir(ppy)3 film and PQT-12/Ir(ppy)3 film on Si/SiO2 substrate at different positions. Figure S3: The output characteristics curves of the optoelectronic synaptic transistor in the dark condition with different VG from 0 to -10 V. Figure S4: The EPSC behavior of the optoelectronic synapse. Figure S5: The PPF behavior and the corresponding PPF index curve of the optoelectronic synapse. Figure S6: The EPSC triggered by light pulses under different wavelengths. Figure S7: Energy band diagram and the steady-state PL spectrum of PQT-12/Ir(ppy)3 heterojunction. Figure S8: The transfer characteristics curves and light switch performance of the pure PQT-12 transistor. Figure S9: The EPSC triggered by light pulses under different light intensities. Figure S10: Schematic diagram of the biological memory consolidation process in the human brain. Figure S11: The STM-to-LTM transition triggered by increasing the illumination intensity of light pulses. Figure S12: LTM behavior of the optoelectronic synaptic transistor. Figure S13: The demonstration of high-pass filtering function of the optoelectronic synapse. Figure S14: Optical logic functions on the optoelectronic synapse through the illumination of blue light with different light intensities. [file 7131895.f1.docx]

**Supplementary Materials**

**Retina-Inspired Organic Heterojunction Based Optoelectronic Synapses for Artificial Visual Systems**

Junyao Zhang^1^, Yang Lu^1^, Shilei Dai^1^, Ruizhi Wang^1^, Dandan Hao^1^, Shiqi Zhang^1^, Lize Xiong^2 *^, Jia Huang^1, 2 *^

^1^ Interdisciplinary Materials Research Center, School of Materials Science and Engineering, Shanghai Institute of Intelligent Science and Technology, Tongji University, Shanghai, 201804, P. R. China.

^2^ Translational Research Institute of Brain and Brain-Like Intelligence, Shanghai Fourth People's Hospital Affiliated to Tongji University, Tongji University, Shanghai, 200434, P. R. China.


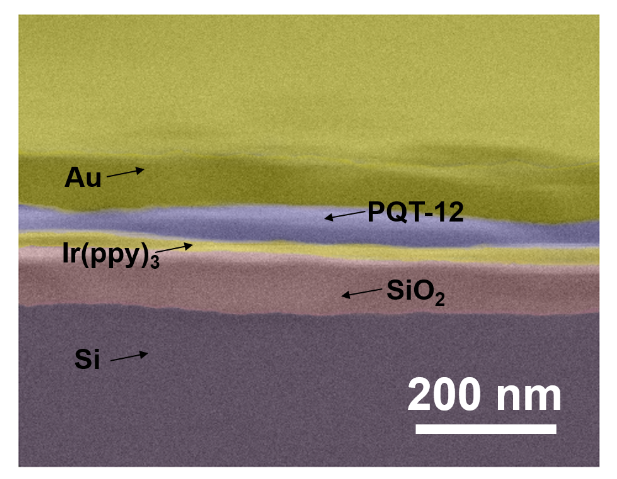


**Figure S1.** Cross-sectional SEM image of the optoelectronic synaptic transistor based on PQT-12/Ir(ppy)_3_ organic heterojunction.


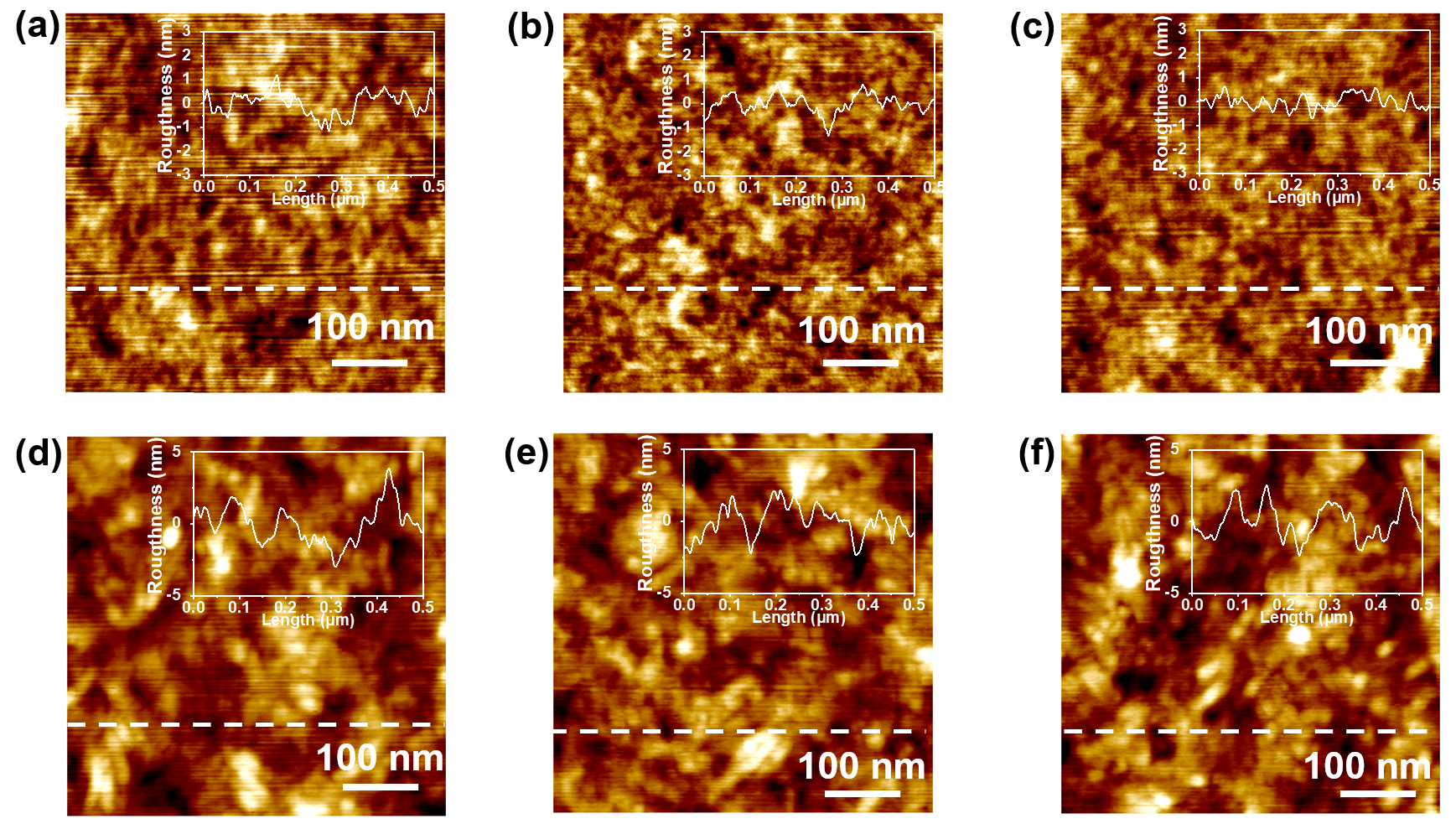


**Figure S2. AFM images of the Ir(ppy)_3_ film and PQT-12/Ir(ppy)_3_ film on Si/SiO_2_ substrate at different positions.** (a-c) AFM images of the Ir(ppy)_3_ film on Si/SiO_2_ substrate at different positions. (d-e) AFM images of the PQT-12/Ir(ppy)_3_ film on Si/SiO_2_ substrate at different positions.


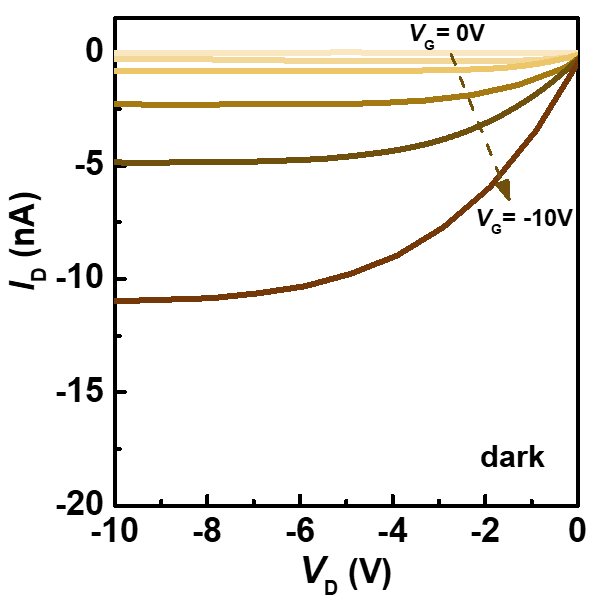


**Figure S3.** The output characteristics curves of the optoelectronic synaptic transistor in the dark condition with different *V*_G_ from 0 to -10 V.


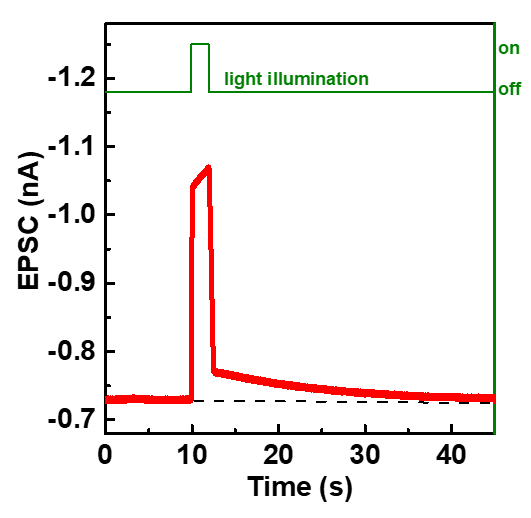


**Figure S4. The EPSC behavior of the optoelectronic synapse.** The EPSC evoked by a light pulse (540 nm, 0.66 mW cm^-2^, 2 s) with a constant *V*_D_ of -2 V and *V*_G_ of 2 V.


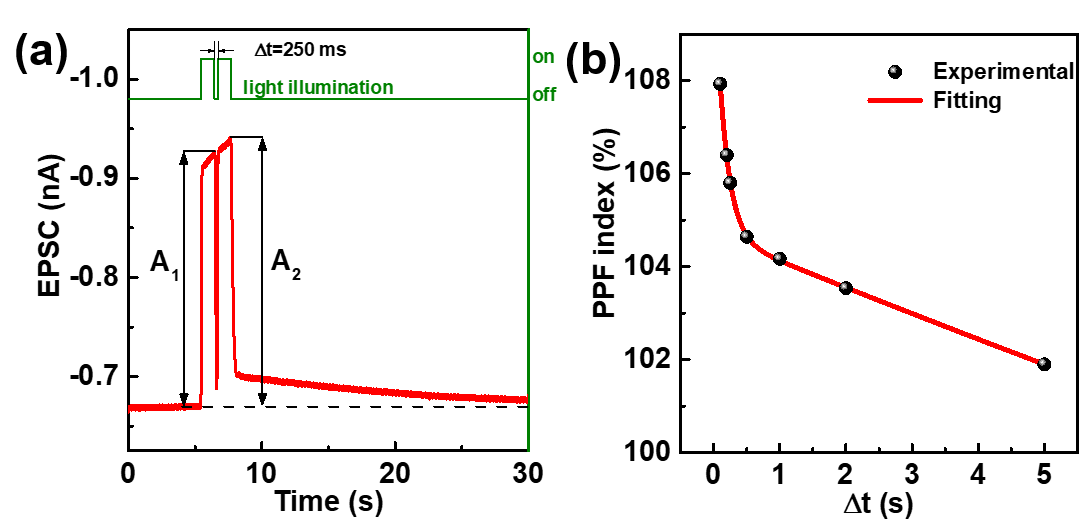


**Figure S5. The PPF behavior and the corresponding PPF index curve of the optoelectronic synapse.** (a) The EPSC evoked by a pair of light pulses (540 nm, 0.66 mW cm^-2^, 2 s) with 250 ms interval at a constant *V*_D_ of −2 V. *A*_1_ and *A*_2_ were the EPSC values at the end of the first- and the second-light pulse, respectively. (b) PPF index (*A*_2_/*A*_1_) as the function of (Δ*t*) with a fixed light pulse intensity of 0.66 mW cm^-2^ and a light pulse duration of 2 s.


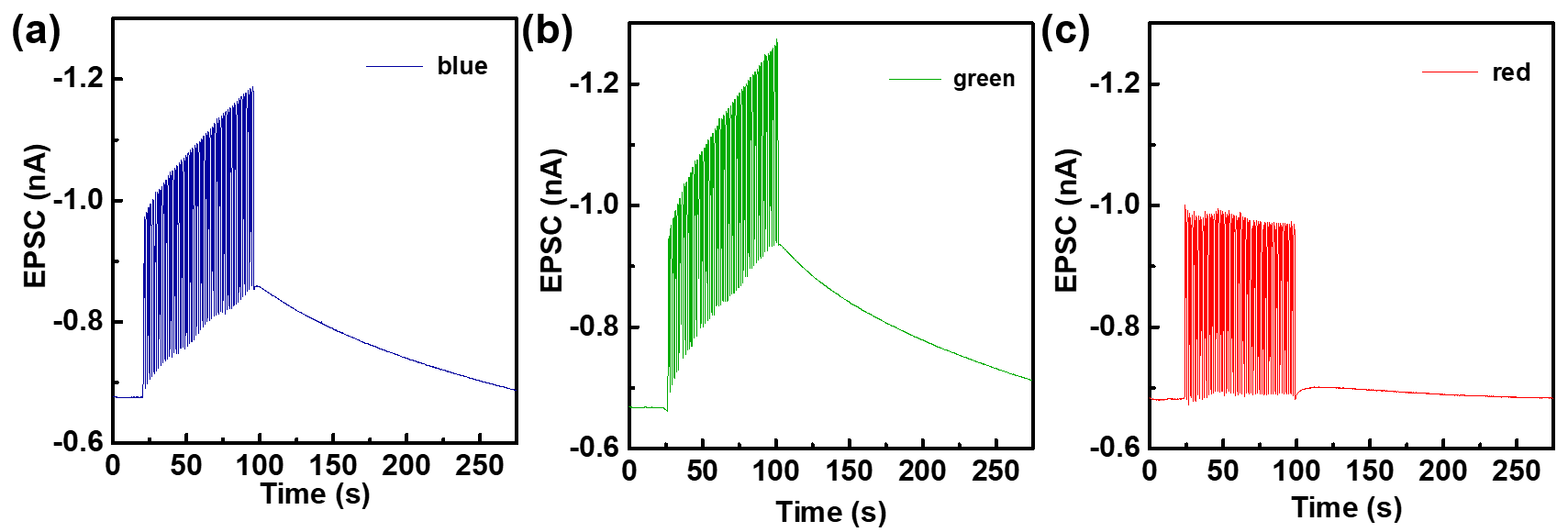


**Figure S6. The EPSC triggered by light pulses under different wavelengths.** The EPSC of the synaptic transistor triggered by 20 successive light pulses under different wavelengths but with a fixed light intensity of 0.66 mW cm^−2^ (1 s duration with 0.5 s interval).


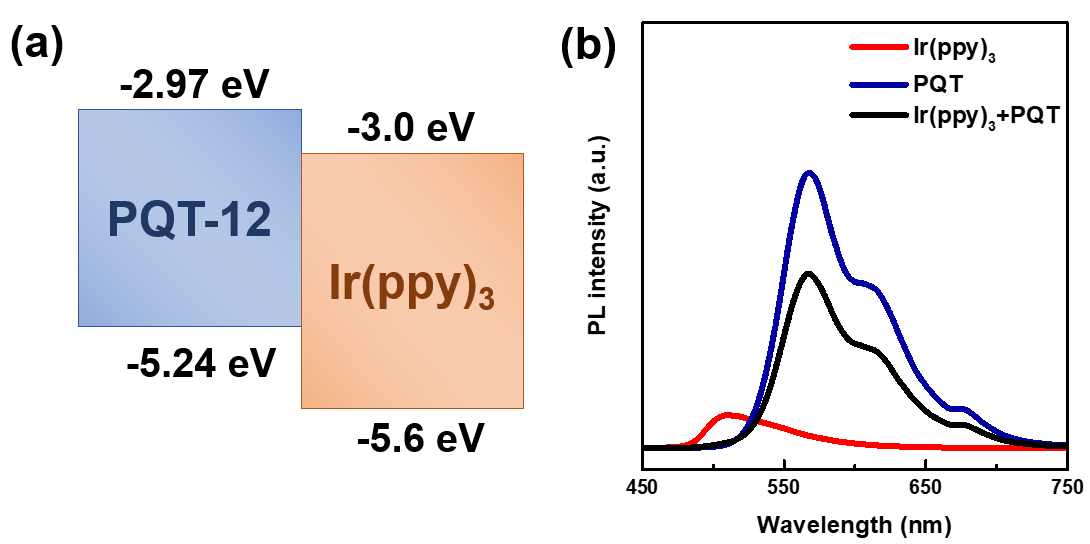


**Figure S7.** **Energy band diagram and the steady-state PL spectrum of PQT-12/Ir(ppy)_3_ heterojunction.** (a) Energy band diagram of PQT-12/Ir(ppy)_3_ heterojunction. (b) The steady-state PL spectrum of the pure Ir(ppy)_3_ thin film, the pure PQT-12 thin film, and the PQT-12/Ir(ppy)_3_ hybrid thin film upon excitation at 380 nm.


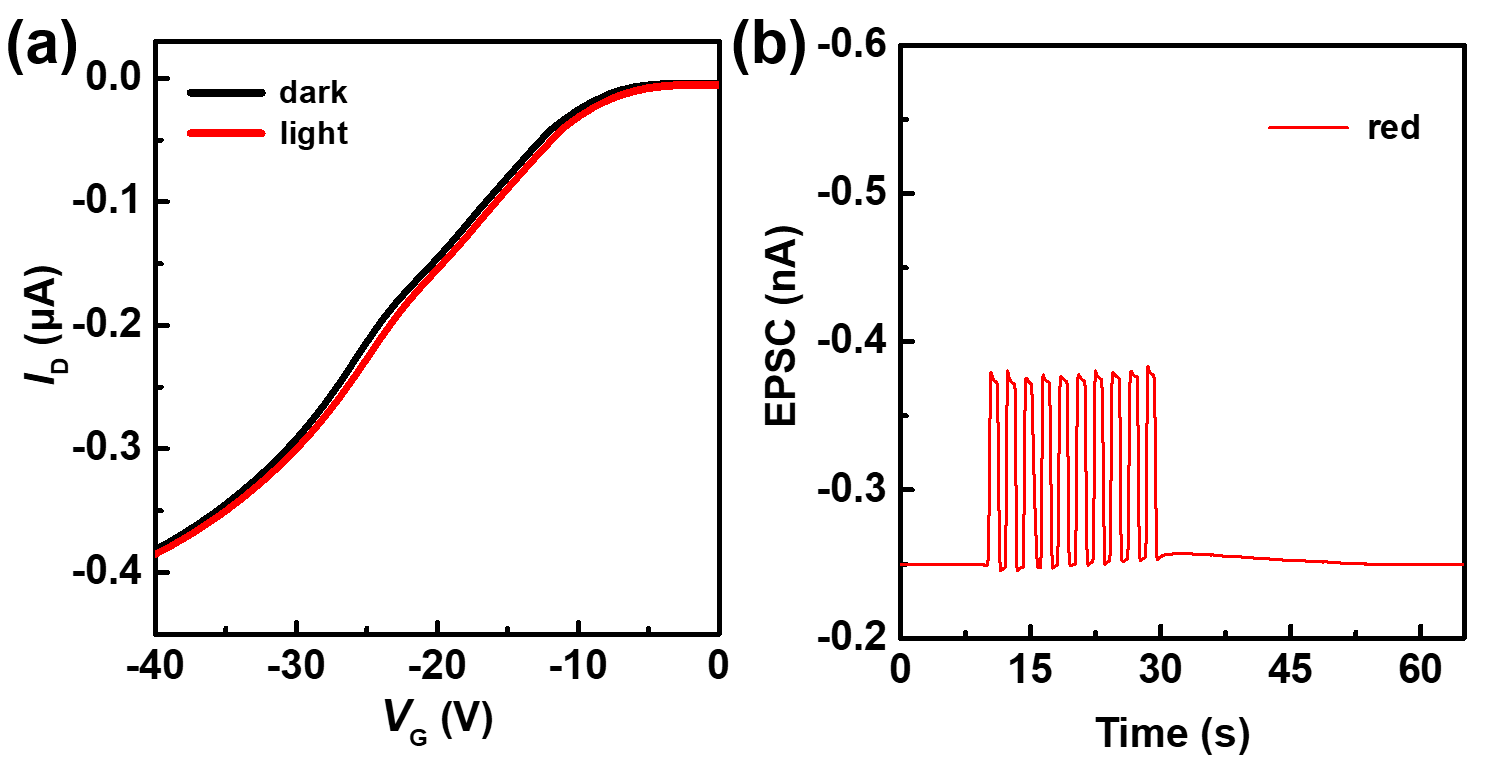


**Figure S8. The transfer characteristics curves and light switch performance of the pure PQT-12 transistor.** (a) Transfer characteristics curves of the pure PQT-12 transistor measured in dark state and under the illumination of fixed wavelength of 540 nm with a constant *V*_D_ of -5 V. (b) Light switch performance of the pure PQT-12 transistor evoked by 10 successive light pulses with a fixed light intensity of 0.66 mW cm^−2^ and a fixed wavelength of 650 nm (1 s duration with 1 s interval).


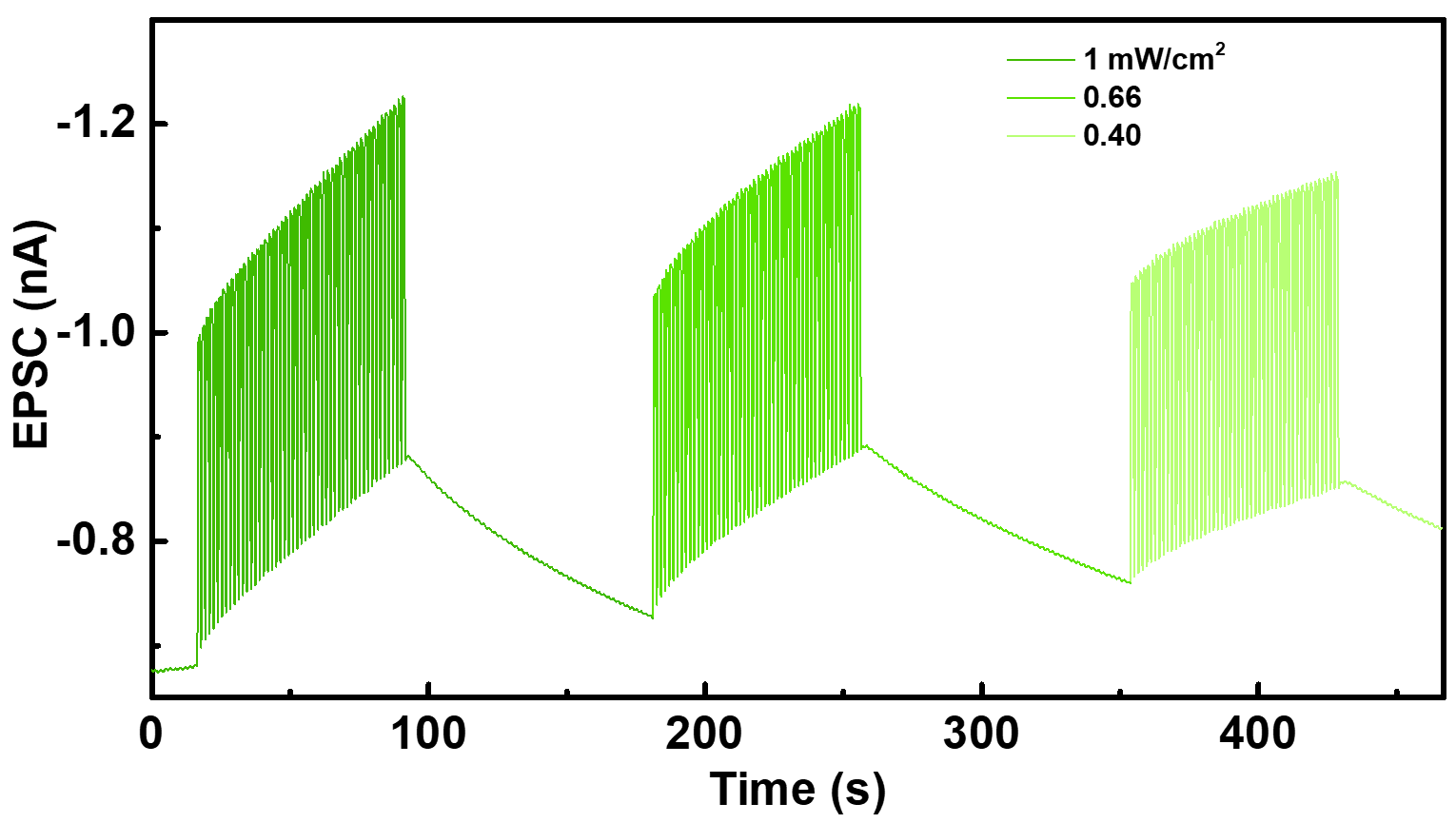


**Figure S9. The EPSC triggered by light pulses under different light intensities.** The EPSC of the synaptic transistor triggered by 20 successive light pulses under different light intensities but with a fixed light wavelength of 540 nm (1 s duration with 0.5 s interval).


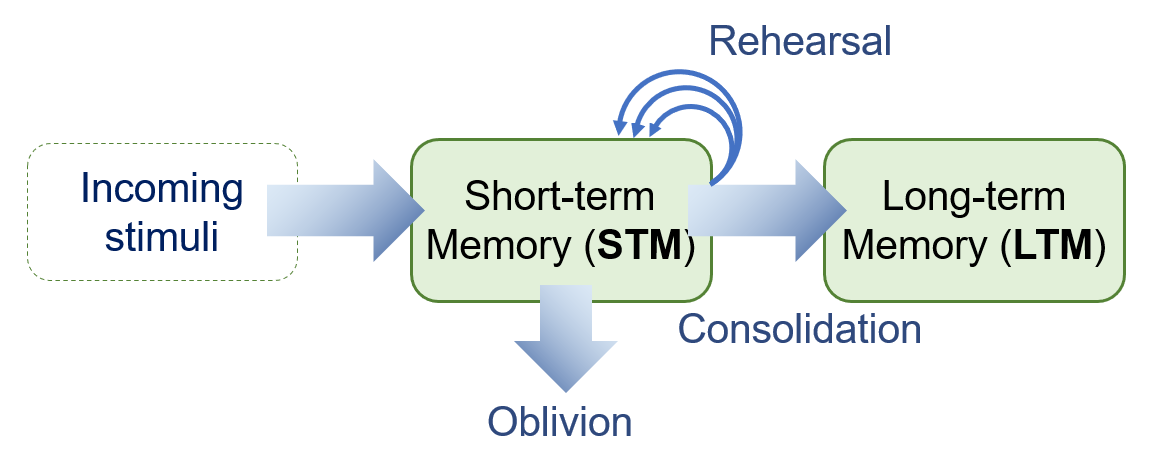


**Figure S10.** Schematic diagram of the biological memory consolidation process in human brain.


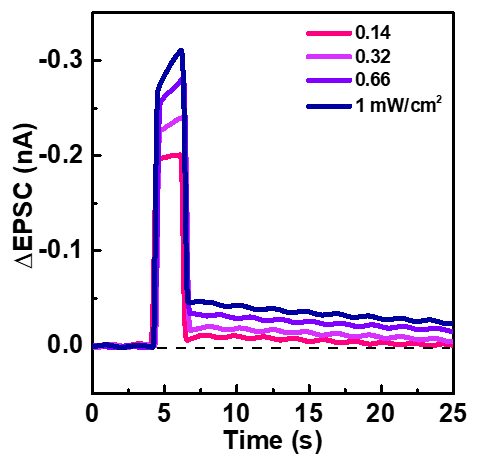


**Figure S11.** The STM-to-LTM transition triggered by increasing the illumination intensity of light pulses.


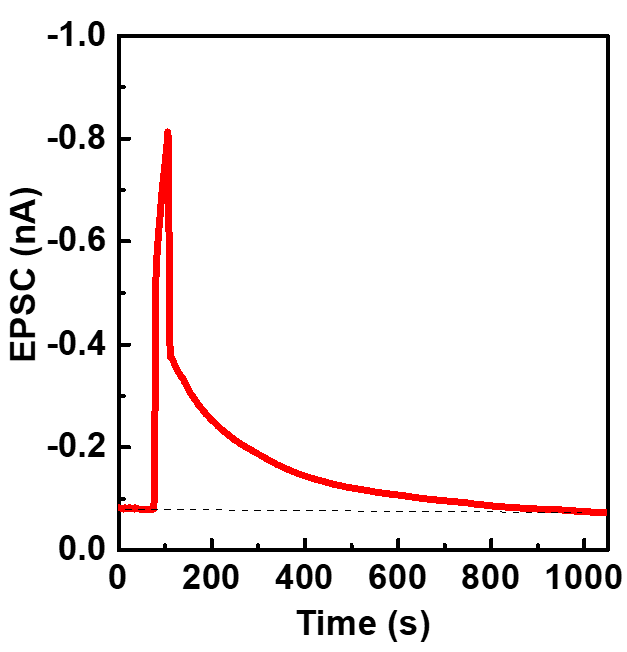


**Figure S12. LTM behavior of the optoelectronic synaptic transistor.** The duration of the light pulse was 30 s (540 nm, 0.75 mW cm^-2^ ) .


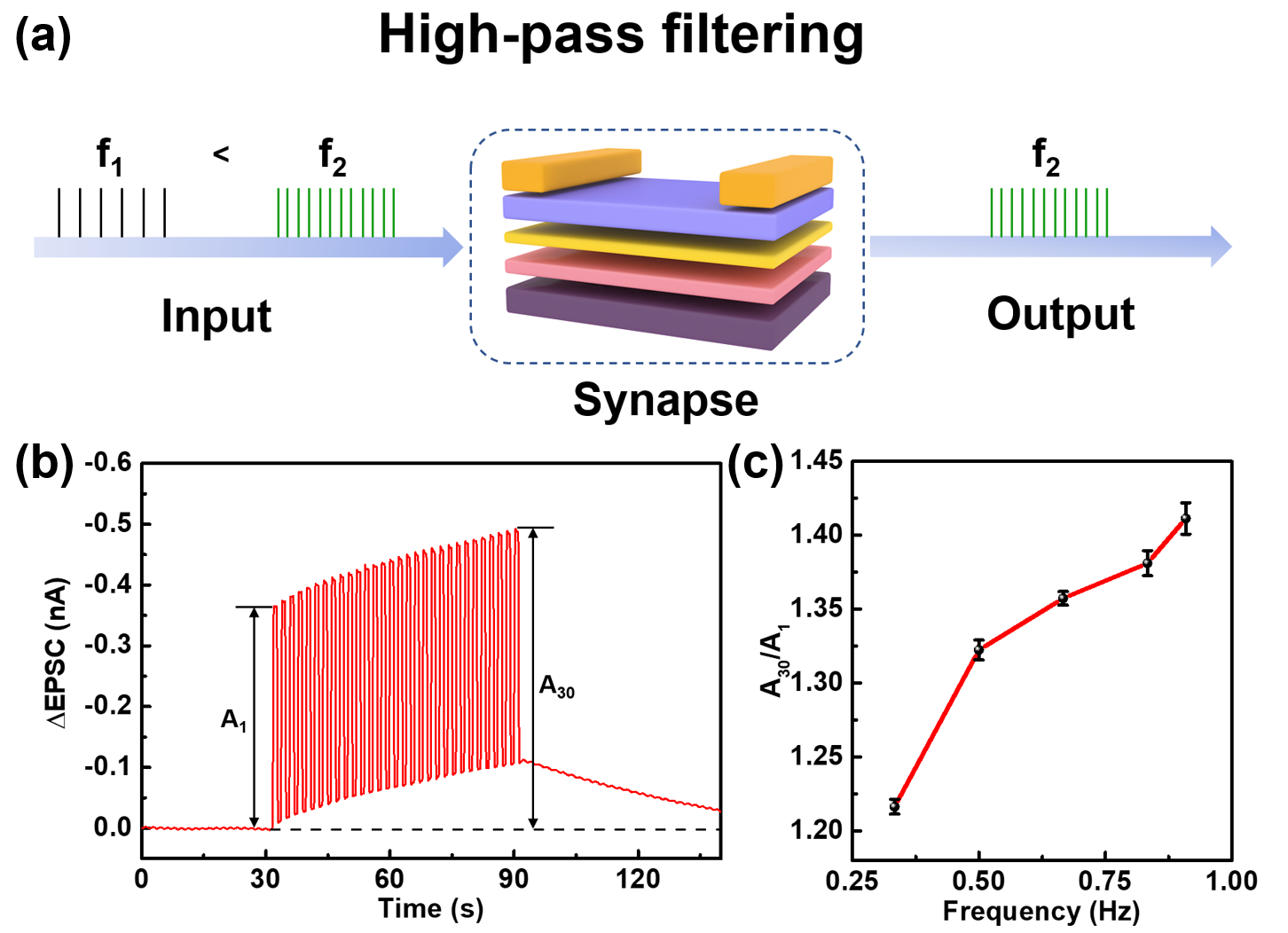


**Figure S13. The demonstration of high-pass filtering function of the optoelectronic synapse.** (a) Schematic diagram of high-pass filtering function. (b) The EPSC of the synaptic transistor triggered by 30 successive light pulses with a fixed light intensity of 0.75 mW cm^−2^ (1 s duration with 1 s interval). *A*_30_ and *A*_1_ represent the amplitudes of the 30th EPSC signal and the first EPSC signal, respectively. (c) EPSC amplitude ratio (*A*_30_/*A*_1_) plotted as a function of the light pulse frequency. Error bars in (c) represented standard errors from three times independent tests of the optoelectronic synapse.


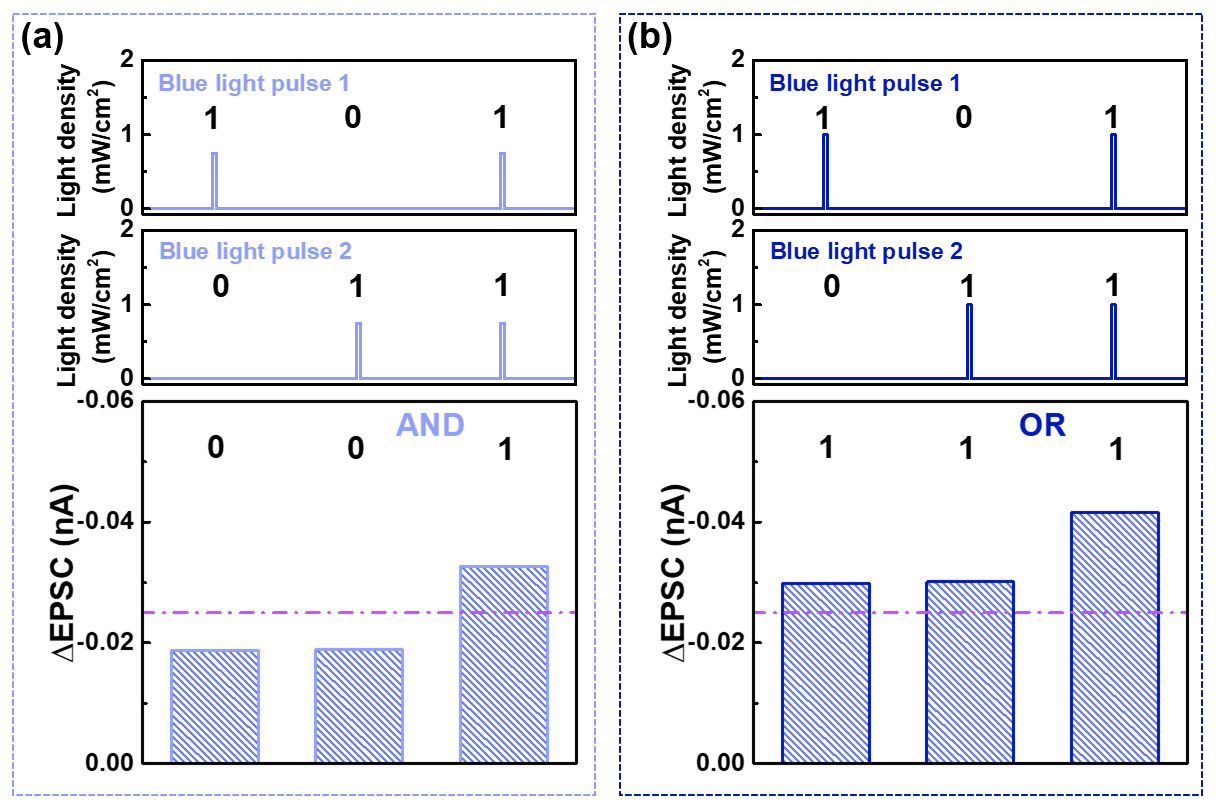


**Figure S14.** **Optical logic functions on the optoelectronic synapse through the illumination of blue light with different light intensities.** The input (presynapse1 (pre 1) and presynapse2 (pre 2)) and output (∆EPSC) characteristics of the (a) “AND” operation and (b) “OR” operation. The duration and wavelength of each light pulse are 1 s and 480 nm, respectively. The purple dash-line represented the threshold of ∆EPSC is set at -0.025 nA.
